# The severity of irritable bowel syndrome or the presence of fibromyalgia influencing the perception of visceral and somatic stimuli

**DOI:** 10.1186/1471-230X-14-182

**Published:** 2014-10-17

**Authors:** Fabrizio Tremolaterra, Serena Gallotta, Yvonne Morra, Ennio Lubrano, Carolina Ciacci, Paola Iovino

**Affiliations:** Digestive Endoscopic Unit, Department of Surgery, A.O.R. “San Carlo”, Via Potito Petrone, 85100 Potenza, Italy; Gastrointestinal Unit, Department of Medicine and Surgery, University of Salerno, Via S. Allende, 84081 Baronissi, SA Italy; Department of Medicine and Health Sciences, University of Molise, Via F. De Sanctis, 86100 Campobasso, Italy

**Keywords:** Adult, Female, Irritable bowel syndrome/complications, Irritable bowel syndrome/physiopathology, Male, Questionnaires, Severity of illness index, Fibromyalgia, Functional bowel disorder severity index (FBDSI)

## Abstract

**Background:**

Fibromyalgia Syndrome (FMS) is a frequent comorbidity in Irritable Bowel Syndrome (IBS) patients with a higher functional bowel disorder severity index (FBDSI). We tested the possibility that mild to severe IBS patients without FMS would have a graduated visceral and somatic perception, and the presence of FMS would further enhance somatic, but conversely attenuate visceral perception.

Our aim was to study visceral and somatic sensitivity in mild IBS patients and in severe IBS patients with or without FMS.

**Methods:**

Eleven mild IBS and 19 severe IBS with and without FMS patients were studied. Somatic and visceral stimuli were applied in each patient by means of electrical stimulations at active and control sites and by means of an electronic barostat in the rectum. Thresholds for discomfort and perception cumulative scores were measured.

**Results:**

Mild and severe IBS patients without FMS demonstrated a significantly lower somatic perception cumulative score than severe IBS patients with FMS at active site. Conversely only severe IBS patients without FMS had significantly lower visceral thresholds for discomfort than mild IBS patients and severe IBS patients with FMS.

**Conclusions:**

The presence of co-existing FMS or greater FBDSI affects somatic and visceral perception in a graded fashion across IBS patients.

## Background

Irritable bowel syndrome (IBS) is a functional intestinal disorder characterized by chronic pain or discomfort in the abdomen associated with altered bowel habits [1]. Patients with IBS often have at least one co-morbid somatic complaint and many IBS patients meet diagnostic criteria for other functional disorders [2, 3]. Interestingly, patients with IBS and another functional disorder, in comparison with patients with only IBS, have more severe IBS symptoms, a higher rate of psychological comorbidity such as depression, anxiety and somatization, greater impairment of quality of life, and more illness-related work absenteeism [4]. In particular, there is a significant association between IBS and fibromyalgia syndrome (FMS) [5, 6]. FMS is chronic non-articular rheumatism where a reproducible physical finding, the presence of tender points, is associated with characteristic symptoms of generalized muscular aches and pains [7]. It has been demonstrated that the association of IBS and FMS depends on the level of severity scored by the functional bowel disorder severity index (FBDSI) more than on the predominant symptom of the intestinal disorder [8, 9]. In this respect, the FBDSI is sensitive enough to distinguish among the different groups, manifesting a logical sequence of illness severity with incremental worsening, from healthy controls through IBS non-patients, to patients with mild IBS only that never showed FMS, and, finally, more severe IBS patients with concomitant FMS [8, 9].

IBS and FMS share important characteristics, including epidemiology (female predominance), pathophysiological hypothesis (inflammation, hypersensitivity, impaired central processing of afferent sensory information, role of serotonin, psychological distress and somatization, and the role of stress and life events), diagnosis (symptom-based), the central role of the patient-physician relationship in therapy, and common therapeutic modalities.

Interestingly, mounting evidence suggests similar changes in pain processing mechanisms. 35–60% of IBS patients had visceral hypersensitivity, whilst studies on somatic pain perception in IBS patients showed contrasting results. Originally IBS patients without FMS showed somatic hyposensitivity at active somatic tender points, non-tender control sites and the T-12 dermatome [10]. Subsequent studies have demonstrated normal skin sensitivity to electrical stimuli at control points [11] or even somatic hyperalgesia with electrical stimuli at active points and areas of pain referral and thermal stimuli on hand and foot. [11, 12]. The coexistence of IBS with FMS that corresponds to a greater illness severity lowered the thresholds to thermal (hand and foot) [13] and electrical stimulation [14] at control (hand and elbow) and active sites (trapezius). Peripheral and central neural dysregulation or both have been involved in explaining these abnormalities in visceral and somatic sensitivity.

In addition, psychological symptoms were shown to contribute to these sensory dysfunctions and may be involved in pain modulation processes that are related to chronic pain [15, 16]. Therefore, this altered sensitivity in IBS patients is consistent with the existence of multiple pathophysiological disorders in IBS patients [17].

Previous studies have reported that the severity of IBS symptoms was positively, although weakly correlated to altered rectal perception [18, 19] and to alteration of pain processes [16]; others failed to find a significant correlation between visceral hypersensitivity and most IBS symptom severity [20], although, many of these studies were limited by the use of non-validated questionnaires. Thus, characterizing the individual patient on the basis of a severity illness instrument already validated would be desirable to overcome the effects of the heterogeneity in pathophysiological mechanisms in IBS [17].

Nowadays, despite the pathophysiological link between IBS and FMS is very interesting and clinically relevant, patients with different IBS severity and the coexistence of FMS have rarely been investigated in the same study to better understand the interactions between both conditions. We tested the possibility that mild to severe IBS patients without FMS would have a graduated visceral and somatic perception, and the presence of FMS co-existing with a more severe IBS would further enhance somatic perception, but conversely attenuate the visceral one.

Our aim was to study visceral and somatic sensitivity in mild IBS patients and in severe IBS patients with or without the coexistence of FMS.

## Methods

### Participants

Thirty IBS patients (19 women, mean age ± SD: 36.4 ± 10.3 yr, range) participated in the study, after giving their informed consent. The study protocol had previously been approved by the medical ethical committee of Federico II University of Naples.

IBS patients were recruited from an outpatients clinic devoted only to functional gastrointestinal disorders (FGIDs). At the same time, two rheumatologists observed all patients recruited for this study. The diagnosis of IBS was made on the basis of the Rome III Criteria [21], together with the exclusion of any organic disease; all patients had an accurate medical history - a physical examination including a rectal examination and routine biochemical tests. Patients with infectious diarrhoea, urological infections, lactose intolerance, parasite infections, helicobacter pylori infection, rectal blood loss, encopresis, neurological or psychiatric diseases and any other organic cause of abdominal pain were excluded from the study.

The severity of IBS was scored using the validated functional bowel severity disorder index (FBSDI) developed by Drossman et al. [22], which provides an easy-to-use scale to appraise illness severity in these patients. In fact, the FBDSI is a measure primarily of pain reporting and behavior. It is comprised of three variables: current pain (by visual analog scale), diagnosis of chronic abdominal pain, and number of physician visits in the past 6 months. Severity is rated as mild (1–36 points), moderate (37–110 points), and severe (≥111 points). Details of the index calculation of the index are presented in the original article [22]. FMS was diagnosed by widespread pain and tenderness in a minimum of 11 of 18 defined tender points (American College of Rheumatology classification criteria) [7]. Patients with signs of inflammatory arthritis documented by clinical, serological and radiological evaluation were excluded. None of the participants were taking serotonin antagonists, pain medications, serotonin uptake inhibitors, or tricyclic antidepressants for at least 3 weeks prior to the study. Subjects were instructed to refrain from the use of any medication for 72 hrs before their sessions. Patients were excluded if they had serious, unstable medical condition, insulin-dependent diabetes mellitus, major psychiatric diagnosis, previous history of drug or alcohol abuse 6 months prior to screening and if they had undergone previous abdominal surgery except appendectomy.

### Somatic perception measurements

Somatic perception was evaluated using a constant current with TENS I (100 Hz, 100 μs) modality through a previously validated Long Stimulus Protocol [14] which consists of intermittent phasic stimuli of 1 mA and 20-s duration and separated by an interval of 30 s from 0 up to a respective threshold for discomfort. Two specific test points were chosen: the non-dominant elbow (the midpoint of the elbow crease in the cubital fossa) as the non-tender point (control site), and the right trapezius (midpoint of the trapezius muscle) as the tender point (active site). At the selected test site, only one somatic stimulus (sham vs electrical stimuli) was given at a time in a random order. In this protocol a sham stimulus consists of a stimulus of 0 mA = no current output and 20-s duration. Subjects were not informed about specific characteristics or the magnitude (mA) of the individual stimuli applied. For somatic perceptions assessment, after each somatic stimulus, the participants were asked to fill in the perception questionnaire.

### Visceral perception measurements

Rectal barostat studies to assess sensitivity were performed as previously reported [23]. Briefly, the barostat maintains a constant pressure on the inside of a bag containing air by means of feedback. The feedback mechanism consists of a strain-gauge connected to an injection/aspiration system by means of a relay. In our study both the strain gauge and the injection/aspiration system were independently connected by a double-lumen polyvinyl tube (12 F, Vygon, Belgium) to a spherical ultra thin bag (capacity 600 ml). Before and after each study, the balloon was checked for leakage. Accordingly to the standardized barostat procedures [24] that advice the cleansing of the studied segment of the gut from any residual content, the evening before the study’s day subjects underwent the application of an enema (sodium diocthylsulfosuccinate/sorbitol, 120 mL) [22]. To our knowledge no potential effects of the chemical substances in the enema have been reported.

After at least a 12 hour fast the carefully folded and lubricated bag was introduced through the anus into the rectum with subjects placed in the left lateral position. A dial allows the selection of the desired pressure level. To unfold the intrarectal bag, one lumen of the polyvinyl tube was connected to a pressure transducer of the barostat device and the bag was slowly inflated through the other lumen of the tube with 200 ml of air under controlled pressure (<20 mmHg). With the balloon inflated, the catheter was pulled back against the pelvic floor and then the bag was completely deflated and connected to the barostat device. Pressure and volume within the bag were continuously recorded. After a 15 minute adaptation period a stepwise protocol (increments in the rectal bag of 1 mmHg and 60 seconds of duration) was selected to measure the minimal distending pressure (MDP) defined as the first pressure inducing an intrarectal volume >30 ml and at which the influence of breathing on the volume was visible [24, 25]. This pressure level accounted for intraabdominal pressure. Afterwards, an intermittent phasic-deflation (distensions) protocol was administered by using the ascending methods of limits with a tracking protocol. Visceral stimuli of 4 mmHg were applied with an interval of 120 seconds, from MDP up to the respective thresholds of discomfort or until the intrabag volume was greater than 550 ml. When the discomfort threshold was reached, a tracking protocol began. The latter consists of successive intermittent phasic distensions occurring in an unpredictable order, at the same or at a lower discomfort pressure, depending on the subject’s responses; in case the distension received a sensation score lower than the discomfort threshold, then the subsequent distention used the same or a higher pressure (in a random order), until the discomfort threshold was reached again. At level at which the patients reported discomfort, the relative pressures were randomly measured three times and then averaged to produce one value.

At each pressure step, intrabag volume was averaged over the last 30 seconds before the next pressure step. The volume-pressure curve was constructed starting from MDP and the rectal compliance (ΔV/ΔP) was used for analysis. During the last 30 seconds of each stimulus the participants were asked to fill in the perception questionnaire.

### Somatic and visceral perception questionnaires

Somatic and visceral perception were tested using graded standardized questionnaires [14, 23, 26]. Shaking, pricking with a needle, tingling and burning were the four sensations included in the somatic perception questionnaire, while the visceral perception questionnaire included bloating, colicky, tenesmus and urge to defecate. Before examinations the questionnaires were fully explained to the participants. The participants were told that after each stimulus the investigator would have ask them to mark in the questionnaire any perceived sensation. Participants were also told, both during somatic and visceral stimulations, to specify any other perceived sensation in an open box in the correspondent questionnaire. Any somatic or visceral sensation was independently evaluated on a graphic rating scale that combines visual descriptors on a visual analog scale graded from 0 to 6. Each participant received standard instructions, specifying that score 0 represented the absence of perception, score 5 represented a sensation of discomfort and score 6 represented a sensation of pain that caused an immediate interruption of the stimulus. Every somatic or visceral sensation was evaluated on the scale, on the basis of its perceived intensity, and orientation descriptors were provided indicating that score 1 represented vague perception of light intensity, score 2 represented definite perception of light intensity, and scores 3 and 4 represented vague and definite perception of moderate sensation, respectively. Participants were also told that if needed, they could indicate half unit scores on the scale, in such a way that scores of intensity were really 12. In each subject the perception score corresponding to each somatic or visceral stimulus was computed and the cumulative score of the common responses was used for comparisons [14, 23, 27]. Discomfort threshold was defined as the first stimulus (electrical stimulus or rectal pressure) that induced a perception score of ≥5. This type of questionnaire has been previously validated in detail by showing its discrimination power and reproducibility in identifying changes in perception in response to increasing stimuli, under conditions that modify perception, and in groups of hypersensitive patients as compared to healthy controls [28, 29].

### Experimental design

Examinations were performed in a quiet, isolated room by the same investigator. Somatic and visceral perception protocols were tested in each patient on different days and in random order, separated by an interval of at least 1 wk.

### Statistical analysis

Data are presented as Mean ± SE, unless otherwise indicated. *χ*^2^ test and analysis of variance (ANOVA) adjusted for age and gender followed by one-way ANOVA for multiple comparisons (Bonferroni)) were used to compare categorical and continuous data, respectively. Spearman correlation test (R) was used when appropriate. Significance was expressed at p < 0.05 level. The SPSS software package for Windows (release 15.0.1; SPSS Inc, Chicago, IL, USA) was used for statistical analysis.

Based on our previous study [14], if the mean difference is 6.7(severe vs mild IBS) and the common within-group standard deviation is 5.2 to find a difference between somatic perception scores using a two-sided test with a significance level of 5% and a power of 80%, it was estimated that a minimum of 10 patients would have been required in each group.

## Results

### Patients

11 mild IBS (6 F, age 35.9 ± 3.0 yrs), 12 severe IBS without FMS (6 F, age 35.2 ± 3.4 yrs), and 7 severe IBS with FMS patients (7 females, age 39.4 ± 3.3), underwent somatic and visceral protocol. Gender and age distribution were not significantly different among groups (*χ*^2^ test, p = 0.09 and Student *t* test, p = 0.7) (Table  1).

**Table 1:** **Demographic variables for IBS patients with and without FMS**

	Mild IBS	Severe IBS without FMS	Severe IBS with FMS	***p***
Patients (n)	11	12	7	
Gender (female), (%)	6 (54)	6 (50)	7 (100)	p = 0.09
Age (mean ± SE)	35.9 ± 3.0	35.2 ± 3.4	39.4 ± 3.3	p = 0.70

### Symptomatic response to somatic stimuli

**Figure 1:**
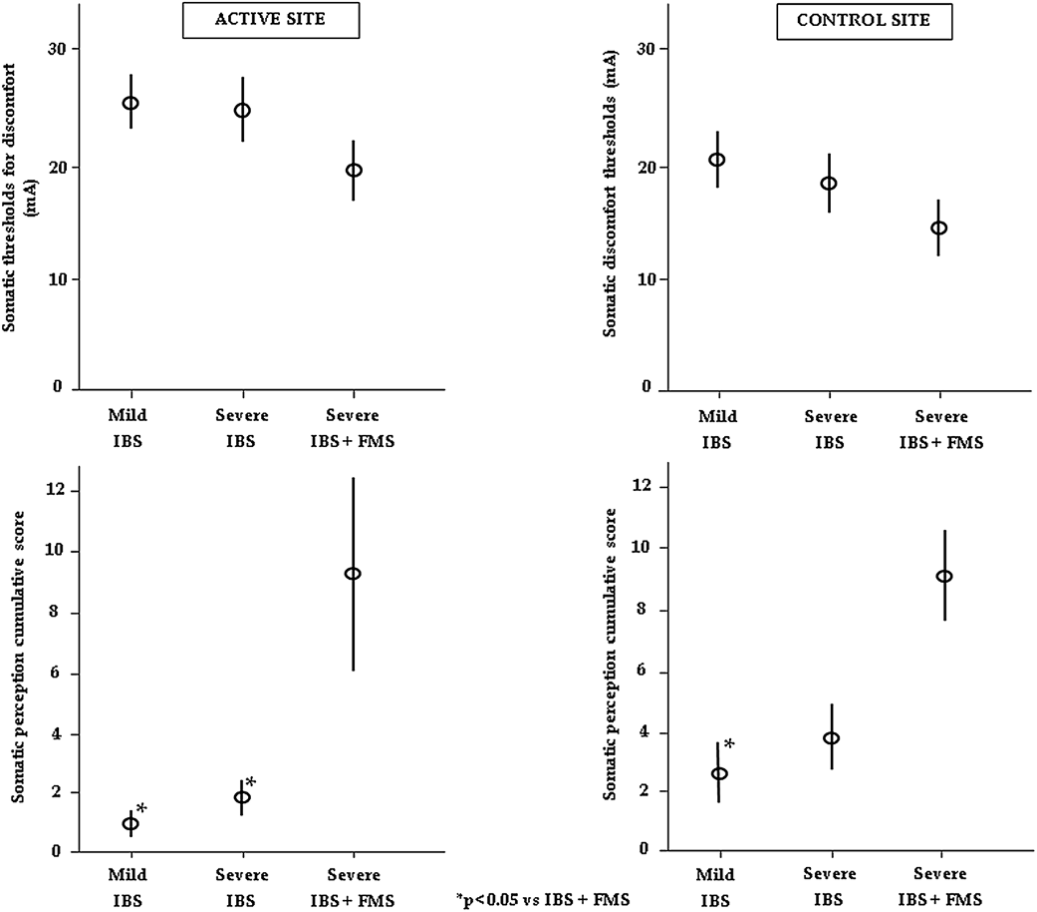
**Somatic thresholds for discomfort did not significantly differ among groups at active and control sites (p = 0.4 and 0.3, respectively).** The somatic perception cumulative score was significantly different among groups at active and control sites (p = 0.006 and p = 0.03, respectively). In detail, at active site mild and severe IBS patients without FMS demonstrated a significantly lower somatic perception cumulative score than severe IBS patients with FMS, whilst at control site only mild IBS patients showed a significantly lower somatic perception cumulative score compared to severe IBS with FMS.

TENS I stimuli induced increased somatic perception in all IBS patients. Thresholds for discomfort did not significantly differ among mild IBS, severe IBS without FMS and severe IBS with FMS at active [25.4 ± 2.2 (16–34) vs 24.9 ± 2.9 (14–44) vs 19.8 ± 2.6 (13–30)] and control sites [20.8 ± 2.4 (11–36) vs 18.6 ± 2.8 (12–35) vs 14.6 ± 2.4 (8–23)] after adjusting for covariates: gender and age (ANOVA, p = 0.4 and 0.3, respectively). Gender and age did not significantly relate to the thresholds for discomfort at active and control sites. There was a lack of significant correlation between IBS severity and somatic thresholds for discomfort (R = -0.286, p = 0.2 at control and R = -0-09, p = 0.7 at active sites, respectively). The somatic perception cumulative score was calculated from 0 to 8 mA, which was used as the upper limit because one patient experienced discomfort at that level. The somatic perception cumulative score was significantly different among groups after adjusting for covariates: gender and age at active site (ANOVA, p = 0.006) and control site (ANOVA, p = 0.03). Gender and age did not significantly relate to the somatic perception cumulative score at active and control sites. At active site mild and severe IBS patients without FMS demonstrated a significantly lower somatic perception cumulative score than severe IBS patients with FMS [1.0 ± 0.4 (0–4) and 1.8 ± 0.6 (0–6) vs 9.3 ± 3.2 (2–24) respectively, m ± SE (min-max), Bonferroni test, p < 0.05], whilst at control site only mild IBS patients without FMS showed a significantly lower somatic perception cumulative score compared to severe IBS with FMS [2.6 ± 1.1(0–10) vs 8.5 ± 1.6(3–14), Bonferroni test p < 0.05] (Figure 1), whilst no significant difference was reached in comparison to severe IBS patients without FMS [3.9 ± 1.2 (0–10)].

### Symptomatic response to visceral stimuli

**Figure 2:**
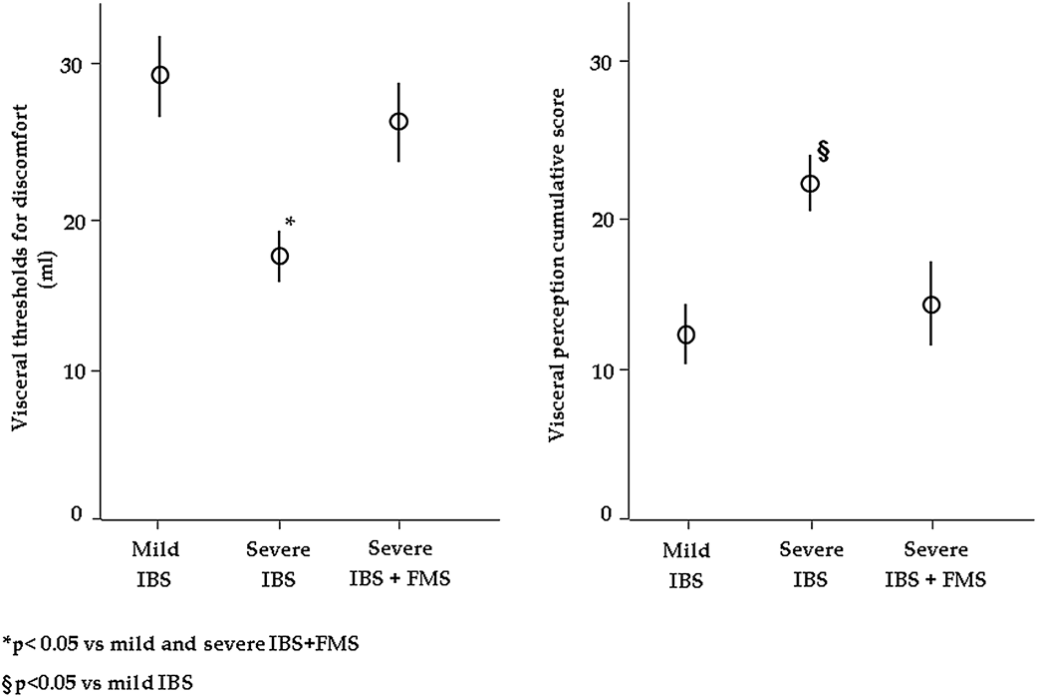
**Visceral thresholds for discomfort were significantly different among groups (p < 0.001).** Severe IBS patients without FMS had significantly lower thresholds for discomfort than mild IBS patients (p < 0.05), and severe IBS patients with FMS (p < 0.05), whilst no differences were found between severe IBS patients with FMS and mild IBS. The visceral perception cumulative score was significantly different among groups (p = 0.03). In detail, severe IBS patients without FMS had a significantly higher visceral perception cumulative score than mild IBS patients (p < 0.05).

Isobaric rectal distensions induced increased perception of symptoms in all IBS patients. Thresholds for discomfort were significantly different among groups after adjusting for covariates: gender and age (ANOVA p < 0.001). Gender and age did not significantly relate to the thresholds for discomfort. In detail, severe IBS patients without FMS had significantly lower thresholds for discomfort than mild IBS patients [17,8 ± 1.4 (12–24) vs 29.3 ± 2.1 (19–37), Bonferroni test p < 0.05], and severe IBS patients with FMS [26.2 ± 2.1(20–35), Bonferroni test p < 0.05], whilst no differences were found between severe IBS patients with FMS and mild IBS.Two patients experienced discomfort at 12 mmHg above MDP, which was used as the upper limit for the calculation of visceral perception cumulative score and compliance. The visceral perception cumulative score was significantly different among groups after adjusting for covariates: gender and age (ANOVA, p = 0.03). Gender and age did not significantly relate to the visceral perception cumulative score. In detail, severe IBS patients without FMS had a significantly higher visceral perception cumulative score than mild IBS patients [23.2 ± 2.9 (11.5-37) vs 12.3 ± 1.98 (2.5-19.5), Bonferroni test p < 0.05] (Figure 2), whilst no significant difference was reached in comparison to severe IBS patients with FMS [14.3 ± 2.8 (5–23)].

### Rectal function

The volume-pressure relationship in response to stepwise increments in intrarectal pressure was linear in IBS patients (individual r from 0.98 to 1.00). MDP was not significantly different in mild IBS, severe IBS and IBS and FMS patients (5.7 ± 1.3 mmHg, 4.8 ± 1.1 mmHg, 4.8 ± 2.1 mmHg respectively, p = 0.9). Rectal compliance was not significantly different among groups.

## Discussion

Visceral hypersensitivity, which is noticeable through reduced threshold for pain, increased intensity of sensations and/or exaggerated viscerosomatic referral in response to colonic distension, is considered to be a biologic marker for IBS and it is found in 35–60% of IBS patients [30, 31].

In numerous IBS studies controlled rectal balloon distension by means of an electronic barostat has been used for reaching the discomfort threshold [18, 23, 24, 28, 29, 32]. A significant correlation between the symptom severity and rectal perception, that is independent from gender, has already been demonstrated in IBS patients [18, 19]. Importantly, the alteration of pain processes is strongly related to IBS symptoms, suggesting that altered pain processes may contribute to the pathophysiology of IBS [16]. To our knowledge, visceral and somatic sensitivity have still not been sufficiently investigated in patients selected for different IBS severity and coexistence of FMS. The results of our study demonstrated that mild and severe IBS patients independent of gender have a graduated visceral sensitivity; however, the presence of FMS that co-exists only with a more severe IBS attenuated visceral sensitivity. Furthermore, the cutaneous sensitivity to electrical stimuli was significantly lower in severe IBS patients with FMS as co-morbidity, whilst it did not significantly increase along with the severity of IBS. No correlation was found between the somatic thresholds for discomfort and the severity of IBS at control and active sites.

It is well known that FMS, an extraintestinal chronic pain disorder characterized by widespread pain, is frequently associated with IBS patients with higher severity of illness [8, 9]. Importantly, somatic mechanical hyperalgesia is a characteristic feature of so-called "tender points" which are a hallmark of FMS and part of the 1990 classification criteria of the American College of Rheumatology for this syndrome [7]. There is a large body of evidence for a generalized lowering of somatic pressure pain thresholds in FMS patients [33, 34], and the mechanical allodynia of FMS patients is not limited to tender points, but appears to be widespread [35]. In addition, almost all studies of FMS patients have shown abnormalities in pain sensitivity while using different methods of somatic sensory testing. It has been previously demonstrated that IBS patients have somatic hypoalgesia to mechanical stimuli [10] and the presence of FMS combined with higher severity of IBS influence the perception of somatic stimuli induced by TENS [14]. Conversely, other studies demonstrated that patients with IBS may also have cutaneous hyperalgesia. Caldarella et al. [11] found in IBS patients a normal skin sensitivity to electrical stimuli, but lowered pain thresholds at the subcutis and muscle when compared to healthy controls (HC); whereas IBS patients with FMS or patients with FMS alone had significantly lower pain thresholds than HC even at skin level. In another study by Moshiree et al., IBS + FMS patients had enhanced thermal sensitivity compared to IBS only patients during foot immersion in hot water [13]. Taking these studies together reinforced the concept that IBS patients may also have somatic hypersensitivity depending on the presence of comorbid FM or greater illness severity [36]. The lack of significant differences in somatic perception between mild and severe IBS patients in this study could be in apparent disagreement with the results of our previous study, in which, however, the presence of FMS could have altered the type of somatosensory perceptual alteration in IBS patients and played a confounding role in the relationships between discomfort thresholds and perception cumulative scores versus IBS severity subgroups [14]. Moreover, it has been already established that differences in testing procedures and stimulus modalities such as mechanical, electrical, thermal and ischemic stimuli could provoke different results in somatic perception. For example, mechanical stimuli did not reveal any evidence of somatic hypersensitivity in either IBS patients or HC [10, 37]. Alternatively, there may be subgroups of IBS patients who differ in their somatic sensitivity in response to different stimuli and, interestingly, among subsets of IBS patients with thermal, ischemic, and cold pressor hypersensitivity a minimal overlap between groups was disclosed [37], suggesting different underlying mechanisms. Less is known about the changes in visceral sensitivity when IBS is associated with FMS. In a previous study IBS and IBS with FMS had significantly lower discomfort thresholds to rectal distention (increased visceral sensitivity) compared to HC, while FMS only patients were normosensitive at the visceral level [11]. One of the major limitations of this study, as enhanced by the same Authors, was the recruitment of mostly severe IBS patients, according to a higher number of positive tender points that should correspond to a higher level of severity [8, 9]. Thus, this group of patients is at a more advanced stage of the disease, which includes some preclinical features of FMS. In this study to overcome this limitation we evaluated mild IBS patients and severe IBS patients with or without concomitant FMS selected using the FBDSI to eventually disclose the differences among groups. The FBDSI is a measure primarily of pain reporting and behavior. It demonstrates known groups’ discriminant validity by differentiating IBS non-patients from IBS patients and IBS patients who also have fibromyalgia corresponding to a greater illness severity of IBS [38]. It has been hypothesized that patients with mild-to-moderate IBS often have more peripherally generated symptoms with gut-based features (i.e., relieved by defecation worse with eating, intermittent, crampy abdominal pain), whereas patients with more severe and painful IBS tend to have more noxious, continuous, and severe symptoms with psychosocial and somatic comorbidities, thus reflecting the greater central nervous system contribution to their illness experience [38]. Furthermore, this concept applies across various medical conditions, such as fibromyalgia, chronic fatigue syndrome, inflammatory bowel disease, or other chronic pain conditions, so much so that when a condition is severe, it is associated with more symptoms of greater intensity [38]. Thus, there is a clinical association of severity with psychosocial and medical comorbidities for these functional conditions. It is beyond the scope of this study to investigate on the multiple pathophysiological link between IBS and FMS. For instance, in both diseases, it has been shown that primary and secondary hyperalgesia are maintained by central sensitization, through tonic nociceptive input from peripheral afferents [39, 40]. In addition, conditioned pain modulation (pain-inhibit-pain mechanisms - CPM) is decreased in both IBS [15, 16, 41] and FMS [42–44]. Moreover, hyperalgesia is associated with decreased CPM in patients with IBS [4], which may result from or contribute to central sensitization, and may predispose to the development of other chronic pain syndromes (e.g. IBS leading to FMS). Another possibility is the contribution of psychological factors to altered pain processing in both diseases.

On the other hand an explanation for graduated hypersensitivity in regard to severity could be the involvement of alternative descending pain-modulatory pathways, which are activated through painful somatic stimuli (due to FMS) and hence attenuate incoming visceral nociceptive information. Conversely, this mechanism can work vice versa for attenuating somatic perception. Another possibility is viscera-somatic conversion at the spinal level [45], which can also explain the observed phenomena.

Given this hypothesis, severity in IBS can be seen as a multi-determined concept that integrates peripheral and central biological processes as they affect symptoms.

### Limitations of the study

There are several limitations in this study. The first is that discomfort threshold was defined as the first stimulus (electrical stimulus or rectal pressure) that induced a perception score of ≥5. One could argue that these are levels at which only low-threshold mechanoreceptors respond with the possibility of having a ceiling effect in our thresholds data. However there are considerable differences in definitions used for discomfort or pain thresholds among laboratories. Moreover, we performed less biased protocols to make the stimulus unpredictable to the subject [46] aiming to reduce psychological bias. However, it is well known that IBS patients may be particularly prone to such bias due to hypervigilance and anticipatory anxiety, even though it is to be established if this anxiety is a cause or consequence of increased visceral perception [47]. Then, the use of standard distension protocols and perception assessment methods in all centres would be desirable.

The second one is the lack of HC to confirm in our patients the expected somatic and visceral hypersensitivity, although the main aim of the study was to evaluate the differences among well selected IBS patients on the basis of illness severity with or without FMS; another limitation is that nearly two-thirds of the study participants were female and no men were present in severe IBS with concomitant FMS. In fact, we were able to enroll a smaller number of patients, only women, with both IBS and FMS due to the known striking prevalence of women in this group [48]. Nevertheless, the impact of gender and age on somatic and visceral perception was taken into account and we demonstrated that was not significantly relevant on our results. This finding confirms previous studies in which the correlation between symptom severity and rectal sensitivity was similar in male and female patients with IBS [18]. Interestingly, differences in brain activation have been reported between men and women with IBS [18, 49, 50], where female patients showed greater activation of regions that could be part of a pain facilitation circuit; whereas male patients showed increased activity in regions that could be involved in pain inhibition. These observations have led some investigators to believe that estrogen is a pronociceptive hormone responsible for the increased pain sensitivity of females. However, this notion has been difficult to prove in women, because the data have generally shown an antinociceptive action of estrogen in both normal females and in those suffering from chronic pain [51–53].

Finally, based on the current design, we were unable to identify the proposed mechanisms that underlie visceral and somatic changes across different IBS severity.

## Conclusion

This study demonstrated a significant association between altered rectal perception and severity of IBS; however, the increase in cutaneous perception to electric stimuli along with the severity of IBS failed to reach statistical significance. The presence of FMS influences both somatic and visceral perception. Although nowadays the exact pathophysiological mechanisms underlying IBS still remain elusive, this study suggests that the IBS patient population may cluster into subgroups that are characterized by different degrees of severity and by the presence of somatic comorbidities such as FMS in which possibly a unique set of pathophysiologic mechanisms are present. Further studies on somatic and visceral sensitivity in IBS patients affected by different degrees of severity and FMS including both a patient group with FMS only and a control group with healthy volunteers are clearly needed for a better pathophysiological understatement and management of these syndromes.

## Abbreviations

FMS: Fibromyalgia syndrome
IBS: Irritable bowel syndrome
FBDSI: Functional bowel disorder severity index
MDP: Minimal distending pressure
HC: Healthy controls.
